# Conventional MRI Features Associated with Histopathological Markers of Biological Aggressiveness in Atypical Meningiomas

**DOI:** 10.3390/jcm15218475

**Published:** 2026-10-30

**Authors:** Hikmet Turan Suslu, Halit Alioglu, Serife Altunbay, Mahmoud Osama, Omar Alomari, Ahmet Rafil Varrak, Zuhal Kuş Silav, Hediye Pınar Gunbey, Mehmet Tiryaki

**Affiliations:** 1 Hamidiye International School of Medicine, University of Health Sciences, Istanbul 34668, Türkiyeahmetrafilvarrak@gmail.com (A.R.V.);; 2 Department of Radiology, Dr. Lutfi Kirdar Kartal Training and Research Hospital, University of Health Sciences, Istanbul 34890, Türkiye; 3 Department of Neurosurgery, Koç University Hospital, Istanbul 34010, Türkiye; 4 Department of Pathology, Dr. Lutfi Kirdar Kartal Training and Research Hospital, University of Health Sciences, Istanbul 34890, Türkiye

**Keywords:** atypical meningioma, magnetic resonance imaging, diffusion-weighted imaging, Ki-67, brain invasion, histopathology

## Abstract

**Background:** Atypical meningiomas (CNS WHO grade 2) are biologically heterogeneous, and reliable preoperative imaging correlates of tumor biology remain incompletely defined. This study investigated associations between routinely acquired conventional MRI features and histopathological markers of biological aggressiveness within this tumor grade. **Methods:** We retrospectively analyzed 90 consecutive patients who underwent surgical resection for histologically defined atypical meningiomas between January 2016 and March 2025. Histopathological specimens were re-reviewed according to the 2021 WHO Classification. Conventional MRI features and apparent diffusion coefficient (ADC) values were evaluated in relation to the Ki-67 labeling index (LI), mitotic count, hypercellularity, spontaneous necrosis, pathological brain invasion, and progesterone receptor expression. Ki-67 LI and ADC were analyzed primarily as continuous variables. A literature-informed Ki-67 threshold of ≥8% was used for secondary categorical analysis, with ≥4% and ≥10% retained as sensitivity analyses. Benjamini–Hochberg false-discovery-rate correction was applied to exploratory categorical comparisons. **Results:** The median age was 59 years, and 56 patients (62.2%) were female. The median Ki-67 LI was 10% (IQR, 5–15), and the median ADC was 0.78 × 10^−3^ mm^2^/s (IQR, 0.73–0.88). ADC showed a weak inverse correlation with Ki-67 LI (ρ = −0.255; 95% CI, −0.44 to −0.05; *p* = 0.015) but was not significantly correlated with mitotic count (ρ = −0.124; *p* = 0.244). In the adjusted continuous model, the association between ADC and Ki-67 was not statistically significant (Ki-67 ratio per 0.1 × 10^−3^ mm^2^/s increase in ADC, 0.95; 95% CI, 0.87–1.04; *p* = 0.264). Similarly, the secondary analysis using Ki-67 ≥ 8% yielded an imprecise association with ADC (OR, 0.865; 95% CI, 0.713–1.002; *p* = 0.054). Among 96 exploratory categorical comparisons, only the association between radiological and pathological brain invasion remained significant after false-discovery-rate correction (q = 0.003). **Conclusions:** Conventional MRI features showed weak and largely exploratory associations with histopathological markers of biological aggressiveness in histologically defined atypical meningiomas. Lower ADC was weakly correlated with higher Ki-67 LI, but this association was not robust after adjustment. Radiological brain invasion was strongly associated with pathological brain invasion. These findings should be considered hypothesis-generating and require prospective multicenter validation incorporating standardized imaging, molecular profiling, and clinically relevant outcomes before clinical application.

## 1. Introduction

Meningiomas are the most common primary intracranial tumors, accounting for approximately one-third of all central nervous system (CNS) neoplasms [1,2,3,4]. While the majority of meningiomas are histologically benign (WHO grade 1), atypical meningiomas (WHO grade 2) comprise about 20–30% of all meningioma cases and demonstrate a significantly higher risk of recurrence and aggressive clinical behavior [5,6]. The 2021 World Health Organization (WHO) Classification further emphasizes the biological heterogeneity of grade 2 meningiomas by integrating histopathological and molecular characteristics into tumor classification, highlighting the need to understand which preoperative imaging findings reflect tumor biology.

Radiological evaluation is central to the preoperative assessment of meningiomas. Conventional MRI and diffusion-weighted imaging are routinely used to characterize tumor size, signal, enhancement, and local invasion [7,8]. Previous studies have associated peritumoral edema, brain or venous sinus invasion, enhancement heterogeneity, and tumor–brain interface irregularity with tumor grade or recurrence [9,10,11,12]. Radiomics and machine-learning approaches may provide additional information, but their clinical use still requires standardized acquisition and external validation [13,14]. Because evidence focused exclusively on grade 2 tumors remains limited, this study examined associations between routinely available MRI features and histopathological markers within a homogeneous cohort of atypical meningiomas.

## 2. Materials and Methods

### 2.1. Study Design and Patient Cohort

This retrospective cohort study included 90 consecutive patients who underwent surgical resection for atypical meningiomas at our institution between January 2016 and 15 March 2025. All histopathological specimens were re-reviewed by an experienced neuropathologist and classified according to the 2021 World Health Organization Classification of Central Nervous System Tumors (5th edition) [15]. TERT promoter mutation and CDKN2A/B homozygous deletion were not routinely assessed during the study period; therefore, tumors were classified according to the histological criteria of the 2021 WHO Classification, and molecularly defined CNS WHO grade 3 tumors cannot be completely excluded. Patients with incomplete clinical data, insufficient preoperative imaging, or inadequate histopathological material for re-evaluation were excluded.

### 2.2. Radiological Evaluation

Preoperative MRI was available for all patients. MRI examinations were performed using two 1.5-T systems: a Magnetom Avanto (Siemens Healthineers, Erlangen, Germany) and a Signa HDxt (GE Healthcare, Milwaukee, WI, USA). Conventional MRI sequences included diffusion-weighted imaging (DWI), T1-weighted imaging, T2-weighted imaging, T2-FLAIR imaging, and contrast-enhanced T1-weighted imaging. DWI was acquired with b-values of 0, 500, and 1000 s/mm^2^ on the Siemens system and 0 and 1000 s/mm^2^ on the GE system. Sequence-specific acquisition parameters for both scanners, including repetition time (TR), echo time (TE), inversion time (TI), matrix, field of view, slice thickness/gap, and number of excitations, are provided in Supplementary Table S1. ADC maps were generated automatically on the scanner console. Gadobutrol (1.0 mmol/mL) was administered intravenously at a dose of 0.2 mmol/kg using a power injector.

Radiological features evaluated included tumor size (<3 cm, 3–6 cm, >6 cm), location, lobulation, T1- and T2-weighted signal intensity, contrast-enhancement pattern, peritumoral edema (PE), tumor–brain interface, vascular flow voids, dural tail sign, bone invasion, venous sinus involvement, and radiological brain invasion. Signal intensity on T1- and T2-weighted images was visually graded using the adjacent temporalis muscle as the reference tissue on a five-point scale, where Grade 1 represented very low signal intensity, Grade 3 represented isointensity with muscle, and Grade 5 represented very high signal intensity, with intermediate grades corresponding to low (Grade 2) and moderately high (Grade 4) signal intensity. The evaluated radiological features were defined according to predefined study criteria as follows:

- Lobulation: Defined as a multilobulated or undulating tumor contour with at least two convex bulges separated by clefts, rather than a smooth round or oval contour.
- Dural tail sign: Defined as linear, tapering dural thickening and enhancement extending from the tumor margin on contrast-enhanced T1-weighted images.
- Vascular flow voids: Defined as curvilinear or punctate signal voids within the tumor or at the tumor–brain interface on T2-weighted images.
- Enhancement pattern: Categorized as homogeneous or heterogeneous enhancement of the solid tumor on contrast-enhanced T1-weighted images.
- Radiological brain invasion: Defined as the presence of one or more of the following findings: loss of the cerebrospinal fluid (CSF)/pial cleft between the tumor and adjacent brain parenchyma, irregularity of the tumor–brain interface with cortical signal abnormalities, or finger-like extensions of high T2/FLAIR signal intensity into the adjacent white matter.
- Venous sinus involvement: Defined as partial or complete tumor invasion of the adjacent dural venous sinus, demonstrated by luminal filling defects, loss of the normal venous flow void, or direct intraluminal tumor extension on contrast-enhanced T1-weighted images.
- Tumor–brain interface: Categorized as smooth/distinct, indicating preservation of a clear CSF/pial plane between the tumor and adjacent brain parenchyma, or irregular/indistinct, indicating partial or complete loss of this interface.
- Peritumoral edema: Graded according to the maximum extent of T2/FLAIR hyperintensity relative to the maximum tumor diameter as follows: Grade 0, no edema; Grade 1, edema extending to less than one-half of the maximum tumor diameter; Grade 2, edema extending from one-half to less than the maximum tumor diameter; and Grade 3, edema extending equal to or greater than the maximum tumor diameter (Figure 1a–c).
- Bone invasion: Defined as cortical erosion, hyperostosis, osteolysis, or direct tumor extension into the adjacent cranial bone on conventional MRI sequences.

Representative examples of the evaluated imaging features are shown in Figure 1, Figure 2 and Figure 3.

All MRI examinations were independently reviewed by two board-certified neuroradiologists who were blinded to the patients’ clinical information and histopathological findings. Any discrepancies between the two readers were resolved by consensus during a joint review session. Apparent diffusion coefficient (ADC) values were quantitatively measured using manually placed regions of interest (ROIs) on ADC maps derived from DWI. ADC measurements were obtained on a single slice using the largest possible elliptical ROI (≥50 mm^2^) placed within the solid enhancing component of the tumor. A single ROI was used for each tumor rather than averaging multiple non-overlapping ROIs. Cystic, necrotic, hemorrhagic, and calcified areas were avoided during ROI placement. Representative ADC measurements are shown in Figure 2.

No radiomic feature extraction, texture analysis, or advanced post-processing techniques were applied. Dynamic contrast-enhanced MRI, diffusion tensor imaging (DTI), arterial spin labeling (ASL), MR spectroscopy, and functional or connectivity imaging were not part of the routine preoperative imaging protocol and were therefore not available for retrospective analysis.

### 2.3. Histopathological Evaluation

All histopathological specimens were re-reviewed by an experienced neuropathologist according to the 2021 WHO Classification of Central Nervous System Tumors (5th edition). Histologically defined CNS WHO grade 2 atypical meningioma was diagnosed in the presence of 4–19 mitotic figures per 10 consecutive high-power fields (HPFs), unequivocal pathological brain invasion, or at least three minor histological criteria. Minor criteria included increased cellularity, small cells with a high nuclear-to-cytoplasmic ratio, prominent nucleoli, sheet-like (patternless) growth, and spontaneous necrosis. Pathological brain invasion was defined as irregular tongue-like or finger-like infiltration of tumor cells into the underlying brain parenchyma beyond the leptomeningeal layer (Figure 4A).

Ki-67 immunoreactivity was evaluated using the MIB-1 clone. The area with the highest density of Ki-67-positive tumor cells (hotspot) was identified by visual assessment at ×400 total magnification. The Ki-67 labeling index was assessed manually by an experienced neuropathologist and expressed as the percentage of Ki-67-positive tumor cells among the evaluated tumor cells. Progesterone receptor (PR) expression was assessed immunohistochemically and recorded as positive or negative as part of the routine pathological evaluation; no predefined quantitative PR scoring threshold was applied. Representative histopathological and immunohistochemical findings, including brain invasion, mitotic activity, spontaneous necrosis, PR expression, and Ki-67 labeling, are shown in Figure 4A–E.

TERT promoter mutation and CDKN2A/B homozygous deletion were not routinely assessed during the study period; therefore, molecularly defined CNS WHO grade 3 tumors cannot be completely excluded.

### 2.4. Statistical Analysis

Statistical analyses were performed using jamovi version 2.7.12.0 and R. Continuous variables were assessed using histograms, Q-Q plots, and the Shapiro–Wilk test. Age, Ki-67 labeling index, mitotic count, and ADC departed from normality and are therefore summarized as median (IQR). Spearman rank correlation was used for continuous associations. Ki-67 was analyzed primarily as a continuous variable; a log-transformed Ki-67 model was used for adjusted analysis with ADC and radiological brain invasion. For the secondary categorical analysis, a literature-informed cutoff of >7.5% was used; because Ki-67 values were recorded as whole percentages, this corresponds to Ki-67 ≥ 8% in the present dataset [16,17]. The ≥4% cutoff and the cohort-median ≥ 10% cutoff were retained as sensitivity analyses. Secondary categorical models used Firth-penalized logistic regression to reduce small-sample bias. ADC was analyzed primarily as a continuous variable; the previously used 0.8 × 10^−3^ mm^2^/s threshold was retained as a secondary analysis [18]. Exploratory categorical associations were tested with Pearson’s chi-square test or Fisher’s exact test as appropriate. Benjamini–Hochberg false-discovery-rate correction was applied across the 96 exploratory comparisons. Two-sided *p* < 0.05 was considered statistically significant.

### 2.5. Ethics Statement

This study was approved by the Institutional Scientific Research Ethics Committee of Kartal Dr. Lutfi Kırdar City Hospital (Approval No: 2025/010.99/17122, dated 25 June 2025). The study was conducted in accordance with the principles of the Declaration of Helsinki. Given the retrospective design, the requirement for informed consent was waived by the ethics committee. Patient confidentiality and anonymity were strictly maintained throughout the study.

## 3. Results

### 3.1. Descriptive Data

A total of 90 patients were included. The median age was 59 years (IQR, 47.3–68.8), and 56 patients (62.2%) were female. Tumor size was available for 88 patients: 13 (14.8%) were <3 cm, 52 (59.1%) were 3–6 cm, and 23 (26.1%) were >6 cm. The median ADC was 0.78 × 10^−3^ mm^2^/s (IQR, 0.73–0.88). The median Ki-67 labeling index was 10% (IQR, 5–15), and 62 tumors (68.9%) had Ki-67 ≥ 8%. The median mitotic count was 5 per 10 HPFs (IQR, 3–7), with 65 tumors (72.2%) meeting the ≥4/10 HPF criterion. Hypercellularity was present in 52 tumors (57.8%), spontaneous necrosis in 50 (55.6%), pathological brain invasion in 56 (62.2%), and progesterone receptor expression was positive in 79 (87.8%) (Table 1).

### 3.2. Correlation Analysis

Spearman rank correlation showed a weak positive association between age and Ki-67 labeling index (ρ = 0.257, *p* = 0.015), a weak inverse association between ADC and Ki-67 (ρ = −0.255, 95% CI −0.44 to −0.05; *p* = 0.015), and a moderate positive association between Ki-67 and mitotic count (ρ = 0.564, 95% CI 0.39–0.70; *p* < 0.001). ADC was not significantly correlated with mitotic count (ρ = −0.124, *p* = 0.244). The continuous relationships are shown in Figure 5 and summarized in Table 2 (Panel A).

### 3.3. Exploratory Categorical Associations

Table 3 presents 96 exploratory categorical comparisons. Ten had nominal *p* < 0.05, but after Benjamini–Hochberg false-discovery-rate correction, only the association between radiological and pathological brain invasion remained statistically significant (q = 0.003). Pathological brain invasion was present in 27 of 29 tumors (93.1%) with radiological brain invasion compared with 29 of 61 tumors (47.5%) without radiological brain invasion. None of the other exploratory associations remained statistically significant after false-discovery-rate correction. Necrosis was not significantly associated with peritumoral edema grade (Fisher’s exact *p* = 0.591).

### 3.4. Continuous and Secondary Ki-67 Analyses

In the adjusted continuous model, each 0.1 × 10^−3^ mm^2^/s increase in ADC was associated with a Ki-67 ratio of 0.95 (95% CI, 0.87–1.04; *p* = 0.264), while radiological brain invasion had a ratio of 0.73 (95% CI, 0.50–1.05; *p* = 0.087) (Table 2 (Panel A)). In the secondary analysis using Ki-67 ≥ 8%, ADC showed an imprecise inverse association (Firth OR per 0.1 × 10^−3^ mm^2^/s increase, 0.865; 95% CI, 0.713–1.002; *p* = 0.054), whereas radiological brain invasion was not associated with high Ki-67 (OR, 0.66; 95% CI, 0.26–1.71; *p* = 0.386) (Table 2 (Panel B)). Analyses using the ≥4% and ≥10% cutoffs were retained as sensitivity analyses (Table 2 (Panel C)).

### 3.5. Radiological Brain Invasion and Ki-67 Sensitivity Analyses

Continuous Ki-67 was modestly lower in tumors with radiological brain invasion (median 8% vs. 10%), but the difference was not statistically significant (*p* = 0.098). The inverse association observed using the ≥4% sensitivity cutoff was markedly attenuated at the literature-informed ≥8% cutoff (OR, 0.66; *p* = 0.386) and remained imprecise at the ≥10% cutoff (OR, 0.48; *p* = 0.099). Within the pathological brain invasion-positive subgroup, Ki-67 was nominally lower in tumors with radiological brain invasion; however, the corresponding penalized estimate was unstable because of sparse data.

## 4. Discussion

Atypical meningiomas exhibit greater proliferative activity and recurrence risk than CNS WHO grade 1 tumors [5,6,19]. Reported recurrence rates underscore the need for reliable markers of tumor biology [20,21,22,23,24,25]. In this cohort restricted to histologically defined CNS WHO grade 2 meningiomas, the associations between conventional MRI features and histopathological markers were generally modest. Lower ADC values showed a weak inverse correlation with the Ki-67 labeling index, whereas no significant correlation was observed between ADC and mitotic count. After adjustment, the association between ADC and continuous Ki-67 was small and not statistically significant, and the secondary analysis using the literature-informed Ki-67 ≥ 8% threshold yielded an imprecise estimate. In contrast, radiological brain invasion showed a strong association with pathological brain invasion and was the only exploratory categorical association that remained significant after correction for multiple testing. These findings suggest that conventional MRI may reflect selected aspects of tumor biology, but its individual features should not be interpreted as stand-alone markers of biological aggressiveness.

Previous imaging studies have associated tumor size, morphology, enhancement characteristics, and diffusion parameters with meningioma grade or outcome [8,11,12,26,27]. Tang et al. reported lower ADC values in higher-grade meningiomas [12], and previous studies have similarly linked reduced ADC with increased cellularity, proliferative activity, and higher tumor grade [12,13,28,29]. A meta-analysis also demonstrated lower ADC values in atypical and anaplastic meningiomas than in benign tumors [13], while radiomic studies have further supported the biological information contained in diffusion-related features [30]. Our findings refine these observations within a cohort restricted to histologically defined grade 2 tumors. Although ADC was weakly correlated with Ki-67, the association was not robust after adjustment and ADC was not significantly correlated with mitotic activity. Thus, ADC may represent a complementary imaging correlate of proliferative activity, but the present data do not support its use as an independent predictor or stand-alone clinical risk marker.

The findings regarding peritumoral edema also warrant cautious interpretation. Tang et al. evaluated edema in relation to meningioma grade [12], whereas our analysis was restricted to grade 2 tumors and used a semi-quantitative grading system based on edema extent relative to tumor diameter. In the revised analysis, edema grade was not significantly associated with continuous Ki-67, and its categorical associations did not remain significant after false-discovery-rate correction. Furthermore, the frequency of spontaneous histopathological necrosis did not differ significantly across edema grades (*p* = 0.591). These findings suggest that the extent of peritumoral edema cannot be explained by proliferative activity or necrosis alone and may also reflect venous, interstitial-fluid, and other microenvironmental mechanisms [31].

Venous sinus involvement remains clinically relevant because of its implications for surgical complexity and recurrence reported in surgical series [29,32,33]. In our cohort, its association with increased mitotic activity was nominal but did not remain significant after correction for multiple comparisons. Accordingly, this finding should be regarded as exploratory rather than evidence that sinus involvement represents a histopathological marker of aggressiveness. Radiological brain invasion, by contrast, showed a strong association with pathological brain invasion, suggesting that conventional MRI can provide useful information regarding tumor–brain interface involvement. Previous imaging studies have similarly examined invasive MRI features in meningiomas [8,12]. However, the inverse association between radiological brain invasion and elevated Ki-67 observed using the ≥4% sensitivity cutoff was not robust. Continuous Ki-67 did not differ significantly according to radiological invasion status, and the association was markedly attenuated when the literature-informed ≥8% threshold was applied. This counterintuitive finding should therefore not be interpreted as a protective biological effect and should not be used for clinical prognostication.

More broadly, most conventional morphological features examined in this study did not retain statistically significant associations with histopathological markers after correction for multiple testing. This is important because several of these features have been associated with aggressive behavior in cohorts containing multiple meningioma grades. Restricting the analysis to grade 2 tumors reduces between-grade biological variation and may consequently diminish the discriminatory value of individual morphological characteristics. Future studies integrating standardized conventional MRI with quantitative imaging, radiomic approaches, molecular profiling, and longitudinal clinical outcomes may better characterize imaging phenotypes within this biologically heterogeneous subgroup [34].

The 2021 WHO Classification incorporates molecular alterations, including TERT promoter mutation and homozygous CDKN2A/B deletion, into meningioma grading. These alterations were not routinely assessed during the study period. Consequently, although all tumors were histologically classified as CNS WHO grade 2, a subset may have fulfilled molecular criteria for CNS WHO grade 3. This potential molecular-grade misclassification may have introduced biological heterogeneity and influenced the observed MRI–histopathology associations. Future studies incorporating systematic molecular characterization are therefore required to determine whether conventional imaging features are associated with specific molecularly defined meningioma subgroups.

This study has several limitations. Its retrospective, single-center design limits causal inference and external generalizability. MRI examinations were performed using two 1.5-T scanners from different vendors with non-identical acquisition protocols, and ADC measurements may be influenced by scanner platform, sequence parameters, and b-value selection. Accordingly, the observed ADC associations and the secondary 0.8 × 10^−3^ mm^2^/s threshold should not be assumed to transfer directly to 3-T systems or other institutions without external validation. Molecular grading markers were not routinely available, introducing the possibility of molecular-grade misclassification. In addition, recurrence/progression dates, follow-up duration, and censoring information were not sufficiently available in the analysis dataset to permit a reliable time-to-event analysis. Finally, the sample size limited multivariable modeling, particularly for dichotomized Ki-67 outcomes. Penalized models were therefore kept parsimonious and interpreted as exploratory association analyses rather than prediction models. Nevertheless, the study provides a systematically reviewed imaging–pathology dataset restricted to histologically defined atypical meningiomas, with blinded radiological assessment, histopathological re-review, and analyses accounting for multiple exploratory comparisons.

## 5. Conclusions

In this single-center cohort of histologically defined atypical meningiomas, conventional MRI features showed weak and largely exploratory associations with histopathological markers of aggressiveness. Lower ADC values were weakly correlated with higher Ki-67 labeling indices but were not associated with mitotic activity, and the adjusted association between ADC and proliferation was small and imprecise. Radiological brain invasion was strongly associated with pathological brain invasion, whereas its inverse association with Ki-67 was not robust across analyses. These findings should be considered hypothesis-generating and should not be used for individual clinical prognostication without prospective multicenter validation incorporating standardized imaging, molecular profiling, and clinically relevant outcomes.

## Figures and Tables

**Figure 1:**
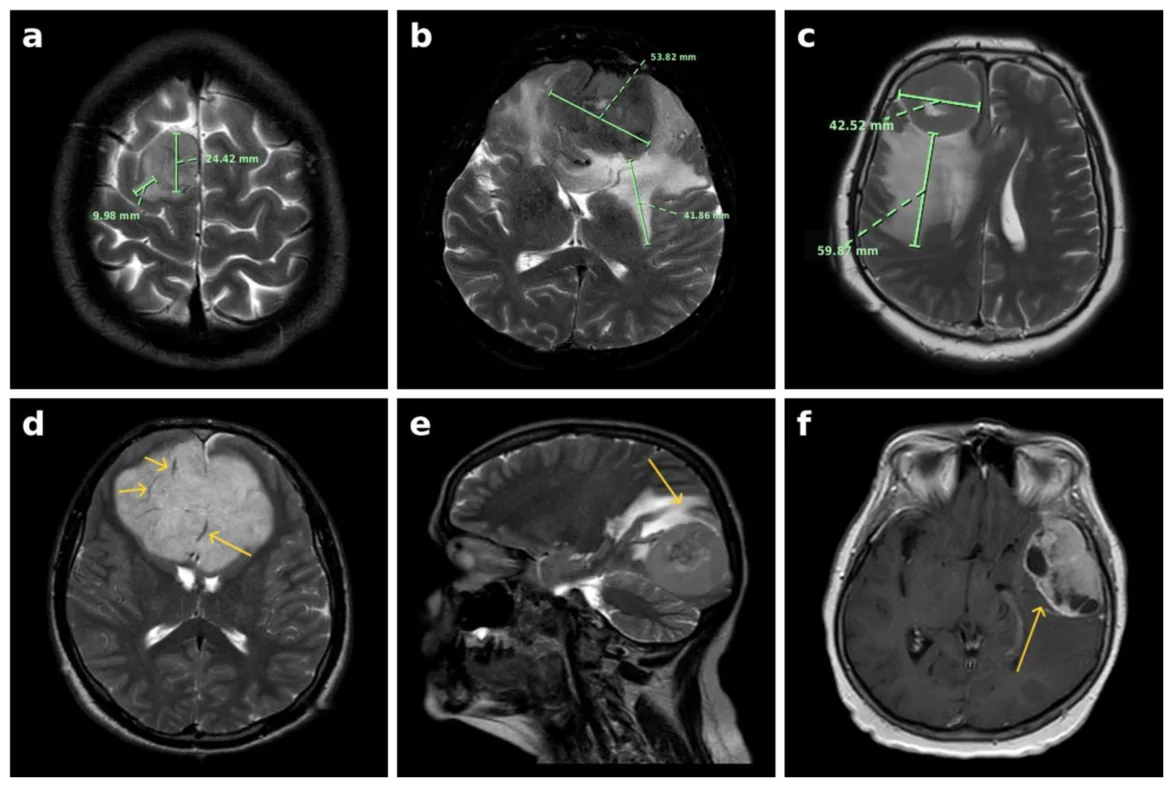
Representative conventional MRI features evaluated in atypical meningiomas. (**a**–**c**) Axial T2-weighted images illustrating the grading of peritumoral edema according to the maximum extent of T2 hyperintensity relative to the maximum tumor diameter. (**a**) Grade 1 in a 36-year-old man: edema extends 9.98 mm compared with a tumor diameter of 24.42 mm (ratio, 0.41). (**b**) Grade 2 in a 67-year-old woman: edema extends 41.86 mm compared with a tumor diameter of 53.82 mm (ratio, 0.78). (**c**) Grade 3 in a 59-year-old man: edema extends 59.87 mm compared with a tumor diameter of 42.52 mm (ratio, 1.41). (**d**) Axial T2-weighted image of a 34-year-old man shows a large lobulated bifrontal mass extending to both sides of the falx, with multiple intratumoral vascular flow voids (arrows). (**e**) Sagittal T2-weighted image of a 50-year-old woman with an occipital mass shows loss of the CSF/pial cleft and a non-discernible cortical margin at the tumor–brain interface (arrow), consistent with radiological brain invasion, accompanied by peritumoral edema. (**f**) Axial contrast-enhanced T1-weighted image of a 57-year-old woman shows a left temporal mass (arrow) with heterogeneous enhancement and cystic-necrotic components. CSF, cerebrospinal fluid.

**Figure 2:**
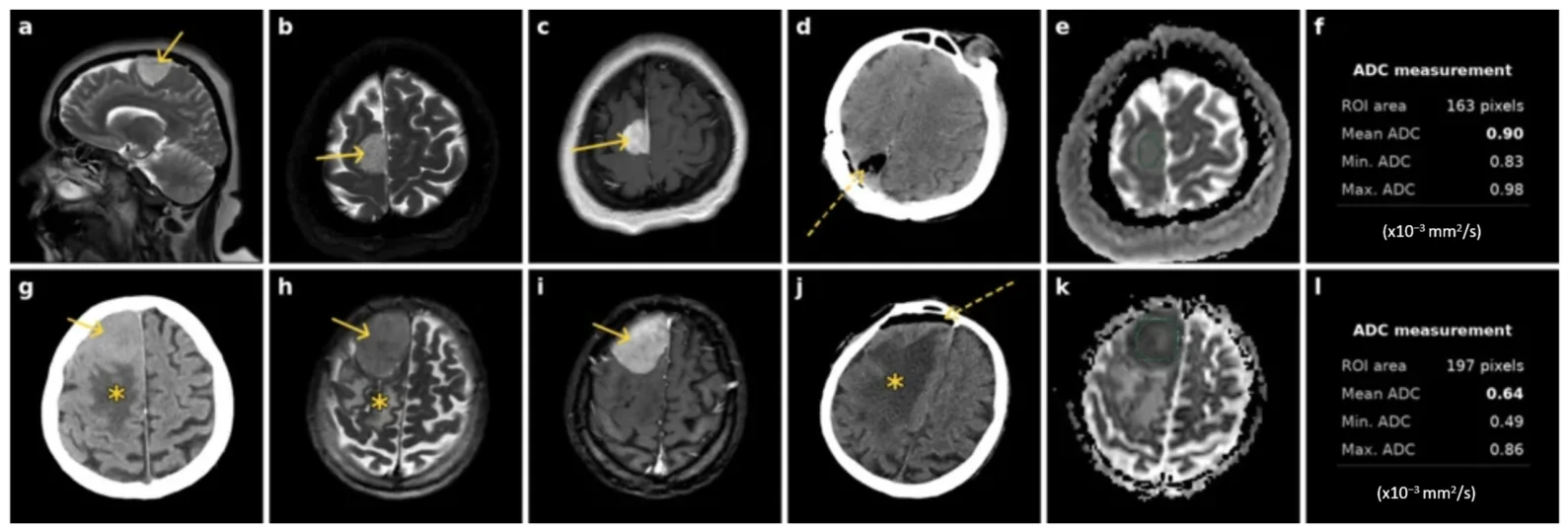
Representative imaging findings in two patients with atypical meningioma (CNS WHO grade 2). (**a**–**f**) Case 1: a 50-year-old woman with a parafalcine meningioma at the vertex. (**a**) Preoperative sagittal T2-weighted and (**b**) axial T2-weighted images show a well-circumscribed extra-axial mass (arrows) without peritumoral edema (grade 0). (**c**) Axial contrast-enhanced T1-weighted image shows intense homogeneous enhancement of the mass (arrow). (**d**) Axial CT obtained on postoperative day 1 shows air in the resection cavity (dashed arrow). (**e**,**f**) ADC map with the region of interest placed within the solid tumor, showing a mean ADC value of 0.90 × 10^−3^ mm^2^/s. The Ki-67 labeling index was 2%, and the tumor was progesterone receptor-positive. (**g**–**l**) Case 2: a 78-year-old man with a right frontal parafalcine meningioma. (**g**) Preoperative axial CT and (**h**) axial T2-weighted image show the extra-axial mass (arrows) with extensive peritumoral edema (asterisks) extending beyond the maximum tumor diameter (grade 3). (**i**) Axial contrast-enhanced T1-weighted image shows avid enhancement of the mass (arrow). (**j**) Axial CT obtained on postoperative day 1 shows postoperative air beneath the frontal bone (dashed arrow) and persistent peritumoral edema (asterisk). (**k**,**l**) ADC map with the region of interest placed within the solid tumor, showing a mean ADC value of 0.64 × 10^−3^ mm^2^/s. The Ki-67 labeling index was 9%, and the tumor was progesterone receptor-positive. ADC, apparent diffusion coefficient; CT, computed tomography.

**Figure 3:**
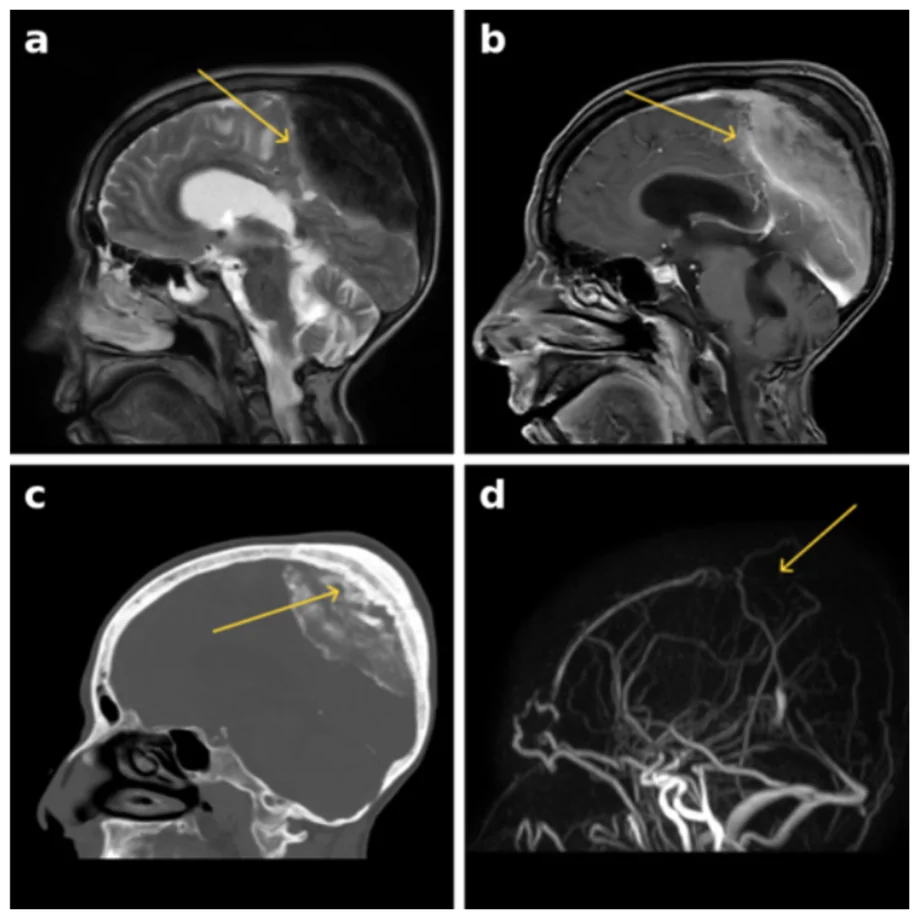
Imaging findings in a 52-year-old woman with a parieto-occipital parasagittal atypical meningioma. (**a**) Sagittal T2-weighted image shows a markedly hypointense mass, consistent with calcification, causing outward protrusion of the scalp and thickening of the adjacent calvarium; the tumor–brain interface is indistinct in places (arrow). (**b**) Sagittal contrast-enhanced T1-weighted image shows enhancement of the mass, with ill-defined enhancement extending into the adjacent brain parenchyma, suggestive of brain invasion (arrow). (**c**) Sagittal non-contrast CT in bone window shows diffuse calcification of the mass and hyperostosis of the adjacent parietal bone (arrow). (**d**) MR venography shows non-visualization of the posterior segment of the superior sagittal sinus at the level of the tumor, with abrupt termination of the sinus in its mid-posterior portion (arrow), consistent with venous sinus involvement. CT, computed tomography; MR, magnetic resonance.

**Figure 4:**
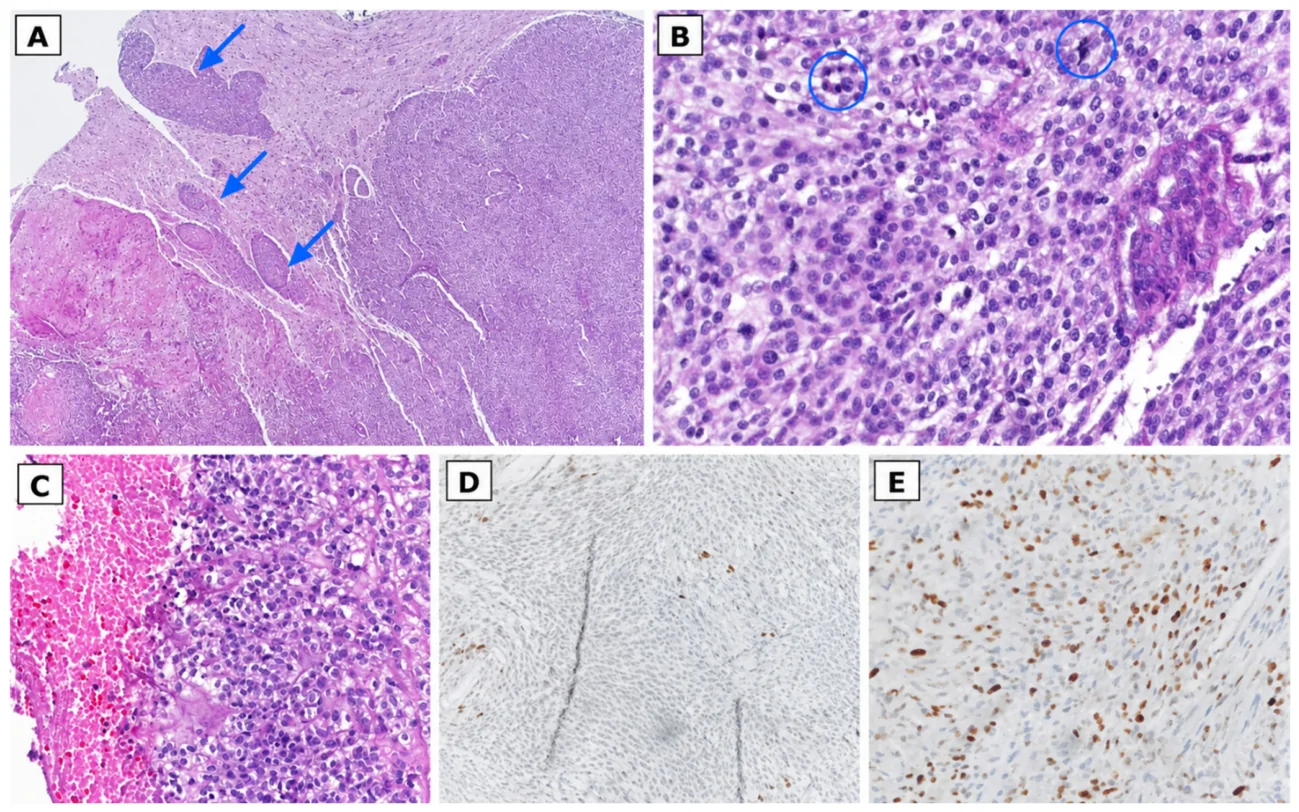
Representative histopathological and immunohistochemical features of atypical meningiomas. (**A**) Finger-like infiltration of tumor cells into the brain parenchyma (arrows), demonstrating pathological brain invasion (H&E, ×40). (**B**) Increased mitotic activity, with two representative mitotic figures indicated by circles (H&E, ×400). (**C**) Atypical meningioma showing spontaneous necrosis on the left side (H&E, ×400). (**D**) Low progesterone receptor (PR) expression (PR immunohistochemistry, ×200). (**E**) High Ki-67 proliferative activity (Ki-67 immunohistochemistry, ×400).

**Figure 5:**
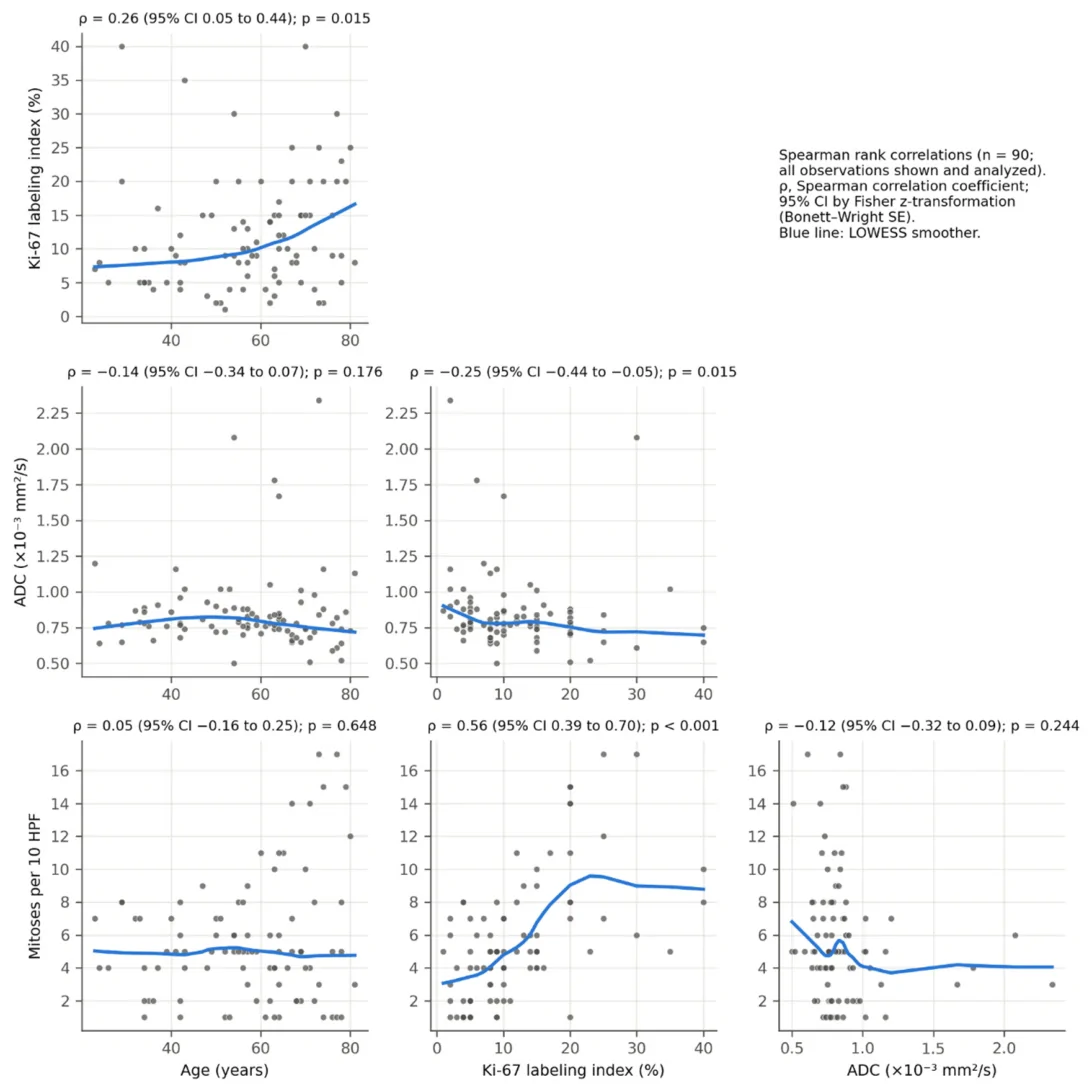
Pairwise Spearman correlations among age, Ki-67 labeling index, apparent diffusion coefficient (ADC), and mitotic count (*n* = 90). Each panel shows the Spearman correlation coefficient (ρ), 95% confidence interval, and *p*-value. The blue line represents a LOWESS smoother.

**Table 1:** Demographic, radiological, and histopathological characteristics of the cohort (*n* = 90).

Characteristic	Cohort Value, *n* (%) or Median (IQR)
Demographic Characteristics	
Age, years, median (IQR)	59 (47.3–68.8)
Female sex	56 (62.2)
Conventional & Quantitative MRI Findings	
Tumor size, cm (*n* = 88) ^a^	
<3 cm	13 (14.8)
3–6 cm	52 (59.1)
>6 cm	23 (26.1)
Tumor location code 1 ^c^	19 (21.1)
Lobulation present	81 (90.0)
Heterogeneous contrast enhancement	32 (35.6)
T1 signal intensity grade	
Grade II (hypointense)	15 (16.7)
Grade III (isointense)	75 (83.3)
T2 signal intensity grade	
Grade I (markedly hypointense)	2 (2.2)
Grade II (hypointense)	3 (3.3)
Grade III (isointense)	40 (44.4)
Grade IV (hyperintense)	38 (42.2)
Grade V (markedly hyperintense)	7 (7.8)
Vascular flow voids present	62 (68.9)
Smooth/distinct tumor–brain interface	60 (66.7)
Dural tail sign present	84 (93.3)
Bone invasion	16 (17.8)
Venous sinus involvement	19 (21.1)
Radiological brain invasion	29 (32.2)
Peritumoral edema grade ^b^	
Grade 0 (absent)	17 (18.9)
Grade I (mild,)	57 (63.3)
Grade II (moderate,)	8 (8.9)
Grade III (severe,)	8 (8.9)
Apparent diffusion coefficient (ADC), ×10^−3^ mm^2^/s, median (IQR)	0.78 (0.73–0.88)
Low ADC (<0.8 × 10^−3^ mm^2^/s)	48 (53.3)
Histopathological & Immunohistochemical Features	
Ki-67 labeling index (LI), %, median (IQR)	10 (5–15)
Ki-67 LI threshold cutoffs	
≥4%	82 (91.1)
≥8%	62 (68.9)
≥10%	46 (51.1)
Mitotic count, mitoses/10 HPF, median (IQR)	5 (3–7)
High mitotic count (≥4/10 HPF ^d^)	65 (72.2)
Hypercellularity present	52 (57.8)
Spontaneous tumor necrosis	50 (55.6)
Pathological brain invasion	56 (62.2)
Progesterone receptor (PR) positive	79 (87.8)

Values are *n* (%) unless otherwise specified. Abbreviations: ADC, apparent diffusion coefficient; HPF, high-power fields; IQR, interquartile range; LI, labeling index; MRI, magnetic resonance imaging; PR, progesterone receptor. ^a^ Two patients with unrecorded tumor size were excluded from the size subgroup analysis (*n* = 88). ^b^ Peritumoral edema graded relative to tumor diameter: Grade 0 = absent, Grade I = mild, Grade II = moderate, Grade III = severe. ^c^ Location code 1 denotes parasagittal/falcine location. ^d^ High mitotic count threshold (≥4/10 HPF) corresponds to the WHO histological grading criteria.

**Table 2:** Continuous correlation and penalized regression analyses evaluating ADC, radiological brain invasion, and Ki-67 labeling index (*n* = 90).

**Panel (A): Continuous Ki-67 Primary Analysis (Correlation & Log-Linear Modeling)**			
**Analysis/Specification**	**Predictor Variable**	**Estimate (95% CI)**	***p*** **-Value**
Spearman rank correlation	ADC vs. Ki-67 LI	ρ = −0.25 (−0.44 to −0.05)	0.015
Spearman rank correlation	ADC vs. Mitotic count	ρ = −0.12 (−0.32 to 0.09)	0.244
Spearman rank correlation	Ki-67 LI vs. Mitotic count	ρ = 0.56 (0.39 to 0.70)	<0.001
Multivariable Log-Linear Model ^a^ (Dependent variable: ln[Ki-67 LI], R^2^ = 0.08)	ADC, per 0.1 × 10^−3^ mm^2^/s increase	Ratio 0.95 (0.87–1.04)	0.264
	Radiological brain invasion (Yes vs. No)	Ratio 0.73 (0.50–1.05)	0.087
**Panel (B): Binary Ki-67 ≥ 4% (82 vs. 8) Firth Penalized Logistic Regression (Secondary Analysis)**			
**Model Architecture (Parameters)**	**Predictor Variable**	**Penalized OR (95% CI) ^b^**	***p*** **-Value ^b^**
Model 1: Univariable ADC (2)	ADC, per 0.1 × 10^−3^ mm^2^/s increase	0.828 (0.696–0.976)	0.026
Model 2: Principal Multivariable (3)	ADC, per 0.1 × 10^−3^ mm^2^/s increase	0.837 (0.683–0.998)	0.048
	Radiological brain invasion (Yes vs. No)	0.16 (0.03–0.71)	0.015
Sensitivity Model with Age (4)	ADC, per 0.1 × 10^−3^ mm^2^/s increase	0.841 (0.687–1.005)	0.057
	Radiological brain invasion (Yes vs. No)	0.17 (0.03–0.73)	0.017
	Age, per 10-year increase	1.00 (0.56–1.69)	0.992
Supplementary: Dichotomized ADC (3)	ADC < 0.8 × 10^−3^ mm^2^/s (Yes vs. No)	8.32 (1.58–86.06)	0.011
	Radiological brain invasion (Yes vs. No)	0.12 (0.02–0.56)	0.006
**Panel (C): Alternative Ki-67 Cutoff Thresholds (Model 2 Architecture)**			
**Cutoff Threshold (High vs. Low *n*)**	**Predictor Variable**	**Penalized OR (95% CI) ^b^**	***p*** **-Value ^b^**
≥4% Threshold (82 vs. 8)	ADC, per 0.1 × 10^−3^ mm^2^/s increase	0.837 (0.683–0.998)	0.048
	Radiological brain invasion (Yes vs. No)	0.16 (0.03–0.71)	0.015
≥8% Threshold ^c^ (62 vs. 28)	ADC, per 0.1 × 10^−3^ mm^2^/s increase	0.865 (0.713–1.002)	0.054
	Radiological brain invasion (Yes vs. No)	0.66 (0.26–1.71)	0.386
≥10% Threshold ^d^ (46 vs. 44)	ADC, per 0.1 × 10^−3^ mm^2^/s increase	0.943 (0.791–1.087)	0.427
	Radiological brain invasion (Yes vs. No)	0.48 (0.19–1.15)	0.099

ADC units expressed in ×10^−3^ mm^2^/s; continuous ADC odds ratios (OR) reflect risk change per 0.1-unit increase (OR < 1.0 indicates that higher ADC is associated with lower odds of elevated Ki-67 LI). Abbreviations: ADC, apparent diffusion coefficient; CI, confidence interval; HC3, heteroskedasticity-consistent robust covariance matrix; LI, labeling index; OR, odds ratio. ^a^ Linear regression modeling ln(Ki-67 LI) against continuous ADC and radiological brain invasion (*n* = 90; model R^2^ = 0.08). Multiplicative back-transformed ratio estimates are presented with HC3 robust 95% CIs. ^b^ Firth penalized likelihood ratio test and profile penalized-likelihood 95% CIs (implemented to overcome sparse data bias and event separation in Ki-67 < 4% subgroup). ^c^ Literature-derived cutoff (Ki-67 > 7.5%, derived via Youden index within WHO Grade 2 meningioma cohorts). ^d^ Cohort median cutoff; data-derived threshold not clinically validated. Methodological Note: For methodological comparison, the original unpenalized six-predictor model yielded severe sparse-data bias: OR = 24.65 (95% CI: 1.67–363.65) for ADC < 0.8 and OR = 0.04 (95% CI: 0.005–0.40) for radiological invasion. Apparent discrimination (c-statistic = 0.92) attenuated to 0.82 following bootstrap optimism correction.

**Table 3:** Exploratory univariable associations between clinical/conventional MRI variables and histopathological markers (*n* = 90).

Predictor Variable	Category	*n*	Ki-67 ≥ 4% (*n* = 82)	Hypercellularity (*n* = 52)	Necrosis (*n* = 50)	Mitoses ≥ 4/10 HPF (*n* = 65)	Path. Brain Invasion (*n* = 56)	PR Positive (*n* = 79)
Age, years	<60	46	42 (91.3)	21 (45.7)	24 (52.2)	36 (78.3)	27 (58.7)	41 (89.1)
	≥60	44	40 (90.9)	31 (70.5)	26 (59.1)	29 (65.9)	29 (65.9)	38 (86.4)
Statistical Significance	*p*/*q*		1.000/1.000	*0.017*/*0.237*	0.509/0.903	0.191/0.829	0.480/0.903	0.689/0.903
Sex	Female	56	49 (87.5)	31 (55.4)	26 (46.4)	42 (75.0)	35 (62.5)	48 (85.7)
	Male	34	33 (97.1)	21 (61.8)	24 (70.6)	23 (67.6)	21 (61.8)	31 (91.2)
Statistical Significance	*p*/*q*		0.250/0.829	0.551/0.903	*0.025*/*0.254*	0.450/0.903	0.944/1.000	0.524/0.903
Tumor size ^a^	<3 cm	13	11 (84.6)	6 (46.2)	7 (53.8)	8 (61.5)	9 (69.2)	9 (69.2)
	3–6 cm	52	47 (90.4)	27 (51.9)	31 (59.6)	38 (73.1)	31 (59.6)	48 (92.3)
	>6 cm	23	22 (95.7)	19 (82.6)	11 (47.8)	18 (78.3)	15 (65.2)	21 (91.3)
Statistical Significance	*p*/*q*		0.512/0.903	*0.026*/*0.254*	0.632/0.903	0.559/0.903	0.861/0.973	0.080/0.455
Lobulation	No	9	9 (100.0)	6 (66.7)	5 (55.6)	6 (66.7)	6 (66.7)	8 (88.9)
	Yes	81	73 (90.1)	46 (56.8)	45 (55.6)	59 (72.8)	50 (61.7)	71 (87.7)
Statistical Significance	*p*/*q*		1.000/1.000	0.728/0.903	1.000/1.000	0.704/0.903	1.000/1.000	1.000/1.000
Contrast enhancement	Homogeneous	58	53 (91.4)	31 (53.4)	30 (51.7)	41 (70.7)	36 (62.1)	52 (89.7)
	Heterogeneous	32	29 (90.6)	21 (65.6)	20 (62.5)	24 (75.0)	20 (62.5)	27 (84.4)
Statistical Significance	*p*/*q*		1.000/1.000	0.263/0.841	0.325/0.903	0.662/0.903	0.968/1.000	0.512/0.903
T1 signal grade	Grade II	15	9 (60.0)	6 (40.0)	9 (60.0)	10 (66.7)	7 (46.7)	14 (93.3)
	Grade III	75	73 (97.3)	46 (61.3)	41 (54.7)	55 (73.3)	49 (65.3)	65 (86.7)
Statistical Significance	*p*/*q*		**<0.001/0.009**	0.127/0.676	0.704/0.903	0.753/0.903	0.173/0.829	0.683/0.903
T2 signal grade	Grade I	2	0 (0.0)	0 (0.0)	0 (0.0)	1 (50.0)	2 (100.0)	1 (50.0)
	Grade II	3	3 (100.0)	2 (66.7)	0 (0.0)	3 (100.0)	1 (33.3)	2 (66.7)
	Grade III	40	36 (90.0)	24 (60.0)	25 (62.5)	28 (70.0)	28 (70.0)	36 (90.0)
	Grade IV	38	37 (97.4)	21 (55.3)	23 (60.5)	30 (78.9)	20 (52.6)	33 (86.8)
	Grade V	7	6 (85.7)	5 (71.4)	2 (28.6)	3 (42.9)	5 (71.4)	7 (100.0)
Statistical Significance	*p*/*q*		*0.010*/*0.218*	0.552/0.903	*0.044*/*0.322*	0.205/0.829	0.297/0.903	0.234/0.829
Vascular flow voids	No	28	27 (96.4)	17 (60.7)	15 (53.6)	21 (75.0)	18 (64.3)	24 (85.7)
	Yes	62	55 (88.7)	35 (56.5)	35 (56.5)	44 (71.0)	38 (61.3)	55 (88.7)
Statistical Significance	*p*/*q*		0.427/0.903	0.705/0.903	0.799/0.913	0.693/0.903	0.786/0.909	0.734/0.903
Smooth/distinct interface	No	30	27 (90.0)	20 (66.7)	15 (50.0)	21 (70.0)	20 (66.7)	27 (90.0)
	Yes	60	55 (91.7)	32 (53.3)	35 (58.3)	44 (73.3)	36 (60.0)	52 (86.7)
Statistical Significance	*p*/*q*		1.000/1.000	0.227/0.829	0.453/0.903	0.739/0.903	0.539/0.903	0.746/0.903
Bone invasion	No	74	68 (91.9)	42 (56.8)	41 (55.4)	54 (73.0)	45 (60.8)	66 (89.2)
	Yes	16	14 (87.5)	10 (62.5)	9 (56.2)	11 (68.8)	11 (68.8)	13 (81.2)
Statistical Significance	*p*/*q*		0.629/0.903	0.673/0.903	0.951/1.000	0.763/0.904	0.553/0.903	0.405/0.903
Venous sinus involvement	No	71	65 (91.5)	40 (56.3)	43 (60.6)	47 (66.2)	42 (59.2)	63 (88.7)
	Yes	19	17 (89.5)	12 (63.2)	7 (36.8)	18 (94.7)	14 (73.7)	16 (84.2)
Statistical Significance	*p*/*q*		0.674/0.903	0.593/0.903	0.065/0.413	*0.014*/*0.218*	0.246/0.829	0.694/0.903
ADC < 0.8 × 10^−3^ mm^2^/s	No	42	35 (83.3)	22 (52.4)	24 (57.1)	29 (69.0)	25 (59.5)	36 (85.7)
	Yes	48	47 (97.9)	30 (62.5)	26 (54.2)	36 (75.0)	31 (64.6)	43 (89.6)
Statistical Significance	*p*/*q*		*0.023*/*0.254*	0.332/0.903	0.777/0.909	0.529/0.903	0.621/0.903	0.576/0.903
Peritumoral edema grade	Grade 0	17	16 (94.1)	5 (29.4)	7 (41.2)	13 (76.5)	10 (58.8)	15 (88.2)
	Grade I	57	54 (94.7)	37 (64.9)	33 (57.9)	42 (73.7)	33 (57.9)	53 (93.0)
	Grade II	8	7 (87.5)	5 (62.5)	5 (62.5)	7 (87.5)	6 (75.0)	5 (62.5)
	Grade III	8	5 (62.5)	5 (62.5)	5 (62.5)	3 (37.5)	7 (87.5)	6 (75.0)
Statistical Significance	*p*/*q*		*0.038*/*0.320*	0.081/0.455	0.591/0.903	0.144/0.729	0.385/0.903	*0.040*/*0.320*
Radiological brain invasion	No	61	59 (96.7)	33 (54.1)	33 (54.1)	48 (78.7)	29 (47.5)	54 (88.5)
	Yes	29	23 (79.3)	19 (65.5)	17 (58.6)	17 (58.6)	27 (93.1)	25 (86.2)
Statistical Significance	*p*/*q*		*0.012*/*0.218*	0.305/0.903	0.687/0.903	*0.047*/*0.322*	**<0.001/0.003**	0.741/0.903
Dural tail sign	No	6	3 (50.0)	3 (50.0)	5 (83.3)	3 (50.0)	5 (83.3)	5 (83.3)
	Yes	84	79 (94.0)	49 (58.3)	45 (53.6)	62 (73.8)	51 (60.7)	74 (88.1)
Statistical Significance	*p*/*q*		*0.008*/*0.218*	0.694/0.903	0.221/0.829	0.342/0.903	0.403/0.903	0.554/0.903

Values represent *n* (%) of patients possessing the histopathological marker within each specific clinical/radiological category. Statistical testing: Pearson χ^2^ test was performed when all expected cell counts were ≥5; otherwise, Fisher’s exact test (including r × c contingency tables) was applied. Bold formatting indicates statistical significance following FDR adjustment (*q* < 0.05). Italicized formatting indicates nominal statistical significance (*p* < 0.05, *q* ≥ 0.05). Abbreviations: ADC, apparent diffusion coefficient; HPF, high-power fields; Path., pathological; PR, progesterone receptor. ^a^ Two patients with unrecorded tumor size were excluded from size analysis (*n* = 88).

## Data Availability

The datasets generated during and/or analyzed during the current study are available from the corresponding author on reasonable request.

## Supplementary Materials

The following supporting information can be downloaded at: https://www.mdpi.com/article/10.3390/jcm15218475/s1, Table S1: MRI acquisition parameters of the two 1.5-T scanners used in the study.;
