# Supplementary material for: Conventional MRI Features Associated with Histopathological Markers of Biological Aggressiveness in Atypical Meningiomas
Source: J Clin Med. 2026 Oct 30;15(21):8475. doi: 10.3390/jcm15218475 (PMC13763682; doi:10.3390/jcm15218475)
Supplement: Supplementary file 1 [file jcm-15-08475-s001.zip › jcm-4510938-supplementary.pdf]

Supplementary Table S1. MRI acquisition parameters of the two 1.5-T scanners used in the study.

Sequence (plane)	Technique	TR/TE/TI (ms)	Matrix	FOV (mm)	Thickness/gap (mm)	NEX	Other
**Siemens Magnetom Avanto, 1.5 T (Siemens Healthineers, Erlangen, Germany)**							
T1-weighted (axial)	SE	489/8.7/–	202 × 256	201 × 230	5/0.5	1	–
T2-weighted (axial)	TSE	4500/89/–	224 × 256	201 × 230	5/0.5	1	–
T2-FLAIR (axial)	IR	6000/120/2000	114 × 200	201 × 230	5/1.0	1	–
T2-FLAIR (coronal)	TIRM	10650/90/2662.5	135 × 256	182 × 260	5/1.0	1	ETL 20
DWI (axial) [a]	SS SE-EPI	4500/127/–	130 × 130	230 × 230	5/1.5	1	GRAPPA 2
CE T1-weighted (axial, coronal, sagittal) [c]	SE	663/17/–	168 × 256	201 × 230	5/0.5	1	–
**GE Signa HDxt, 1.5 T (GE Healthcare, Milwaukee, WI, USA)**							
T1-weighted (axial)	FSE	460/8.35/–	256 × 288	240 × 240	5.5/1.5	1	–
T2-weighted (axial)	FSE	5040/124.5/–	224 × 320	240 × 240	5.5/1.5	1	–
T2-weighted (sagittal)	FRFSE	4069/104.3/–	160 × 320	250 × 250	5/1.5	2	ETL 24; ASSET
T2-FLAIR (axial)	FSE-IR	8802/97.1/2193	224 × 320	240 × 240	5.5/1.5	1	–
DWI (axial) [b]	SS SE-EPI	6050/100.3/–	128 × 128	260 × 260	5.5/1.5	2	ASSET
CE T1-weighted (axial, coronal, sagittal) [c]	FSE	460/7.74/–	192 × 256	240 × 240	5.5/1.5	1	–

[a] b = 0, 500 and 1000 s/mm^2; three-scan trace. [b] b = 0 and 1000 s/mm^2. [c] Parameters refer to the axial acquisition. Apparent diffusion coefficient maps were generated automatically on the scanner console. Gadobutrol (1.0 mmol/mL) was administered intravenously at 0.2 mmol/kg with a power injector.

CE, contrast-enhanced; DWI, diffusion-weighted imaging; EPI, echo-planar imaging; ETL, echo train length; FLAIR, fluid-attenuated inversion recovery; FOV, field of view; FRFSE, fast recovery fast spin-echo; FSE, fast spin-echo; IR, inversion recovery; NEX, number of excitations; SE, spin-echo; SS, single-shot; TE, echo time; TI, inversion time; TIRM, turbo inversion recovery magnitude; TR, repetition time; TSE, turbo spin-echo.
